# Development of an Enomogram to Predict the Rate of Loco-Regional Control After Radio-Chemotherapy and Interventional Radiotherapy in Cervical Cancer

**DOI:** 10.3390/cancers18071096

**Published:** 2026-03-27

**Authors:** Valentina Lancellotta, Maria Concetta La Milia, Rosa Autorino, Enrico Rosa, Bruno Fionda, Pierpaolo Dragonetti, Leonardo Bannoni, Raffaella Michela Rinaldi, Viola De Luca, Gerardina Stimato, Angeles Rovirosa, Alessio Giuseppe Morganti, Gabriella Macchia, Benedetta Gui, Nicolò Bizzarri, Anna Fagotti, Luca Tagliaferri, Maria Antonietta Gambacorta

**Affiliations:** 1 Radioterapia Oncologica, Dipartimento di Diagnostica per Immagini e Radioterapia Oncologica, Fondazione Policlinico Universitario “A. Gemelli” IRCCS, 00168 Rome, Italy; mariaconcetta.lamilia@guest.policlinicogemelli.it (M.C.L.M.); rosa.autorino@policlinicogemelli.it (R.A.); bruno.fionda@policlinicogemelli.it (B.F.); pierpaolo.dragonetti@guest.policlinicogemelli.it (P.D.); leonardo.bannoni@policlinicogemelli.it (L.B.); raffaellamichela.rinaldi@guest.policlinicogemelli.it (R.M.R.); viola.deluca@policlinicogemelli.it (V.D.L.); luca.tagliaferri@policlinicogemelli.it (L.T.); mariaantonietta.gambacorta@policlinicogemelli.it (M.A.G.); 2 UOC Fisica per le Scienze della Vita, Dipartimento di Diagnostica per Immagini e Radioterapia Oncologica, Fondazione Policlinico Universitario “A. Gemelli” IRCCS, 00168 Rome, Italy; enrico.rosa@guest.policlinicogemelli.it (E.R.); gerardina.stimato@policlinicogemelli.it (G.S.); 3 Department of Theoretical and Applied Sciences, eCampus University, 22060 Novedrate, Italy; 4 Radiation Oncology Department, Hospital Clínic-Universitat de Barcelona, 08036 Barcelona, Spain; rovirosa@clinic.cat; 5 Radiation Oncology, Department of Medical and Surgical Sciences (DIMEC), IRCCS Azienda Ospedaliero-Universitaria di Bologna, University of Bologna, 40138 Bologna, Italy; alessio.morganti2@unibo.it; 6 Radiation Oncology Unit, Gemelli Molise Hospital, Università Cattolica del Sacro Cuore, 86100 Campobasso, Italy; macchiagabriella@gmail.com; 7 Department of Imaging and Radiation Oncology, Fondazione Policlinico Universitario “A. Gemelli” IRCCS, 00168 Rome, Italy; benedetta.gui@policlinicogemelli.it; 8 Unità Operativa Complessa Ginecologia Oncologica, Dipartimento per la Salute Della Donna e del Bambino e Della Salute Pubblica, Policlinico Agostino Gemelli Istituto di Ricovero e Cura a Carattere Scientifico, 00168 Rome, Italy; nicolo.bizzarri@policlinicogemelli.it (N.B.); anna.fagotti@policlinicogemelli.it (A.F.); 9 Università Cattolica del Sacro Cuore Sede di Roma, 00168 Rome, Italy

**Keywords:** cervical cancer, magnetic resonance imaging, radiotherapy

## Abstract

**Objective**: This study aimed to explore the association between magnetic resonance imaging (MRI)-derived volumetric parameters and oncological outcomes, and to develop an exploratory predictive model based on these variables in patients treated with radio-chemotherapy followed by interventional radiotherapy (modern brachytherapy). **Methods**: Between 2021 and 2024, 300 patients with cervical cancer were included. Treatment was pelvic external beam radiotherapy with platinum-based chemotherapy followed by interventional radiotherapy boost. Volumetric MRI variables for each patient were collected. Time-to-event analyses were performed using Cox proportional hazards regression models. Model performance was assessed using Harrell’s concordance index (C-index). Internal validation was performed using bootstrap resampling. Based on the final multivariable Cox models, an interactive web-based nomogram was developed as an exploratory tool to visualize model-derived associations. **Results**: Median tumor volume decreased from 69.4 cm^3^ at diagnosis to 2.2 cm^3^ at the time of pre-interventional radiotherapy MRI, with a median reduction rate of 96.5%. Tumor volume at diagnosis, pre-interventional radiotherapy residual tumor volume, and tumor volume reduction rate were significantly associated with loco-regional relapse and distant metastases in Cox regression analyses. These findings were consistent across univariate and multivariable models. Internal validation confirmed the stability of the model estimates. **Conclusions**: MRI-derived volumetric parameters are associated with oncological outcomes in patients with locally advanced cervical cancer and may contribute to early risk stratification. The proposed model should be considered exploratory and hypothesis-generating and requires external validation before any potential clinical application.

## Simple Summary

Locally advanced cervical cancer is commonly treated with combined radiotherapy and chemotherapy followed by interventional radiotherapy (modern brachytherapy), although individual responses and outcomes remain heterogeneous. The aim of this study was to explore the association between magnetic resonance imaging-derived volumetric parameters and oncological outcomes. By integrating tumor volume at diagnosis, residual tumor volume before interventional radiotherapy, and tumor shrinkage rate, an exploratory predictive model was developed to estimate individual risk of loco-regional relapse and distant metastases. This predictive model may help clinicians stratify patients according to risk, support personalized treatment planning, and guide follow-up intensity based on early imaging response. The study highlights the potential value of imaging-derived biomarkers for improving decision-making and represents a step toward more individualized management strategies in cervical cancer.

## 1. Introduction

Worldwide, cervical cancer ranks second in incidence and mortality among women of reproductive age [1], with the heaviest burden observed in nations with the lowest Human Development Index [2,3].

Pelvic external beam radiotherapy with concurrent platinum-based chemotherapy followed by image-guided interventional radiotherapy (modern brachytherapy) represents the standard treatment for locally advanced cervical cancer [4].

Imaging plays a central role in the management of the disease by assessing local and lymph node extension, evaluating treatment response, and detecting recurrence [5,6]. Assessment of tumor volume changes represents an important parameter. Magnetic resonance imaging (MRI) is the first-line modality for all these indications, with diffusion-weighted sequences providing essential information that benefits both radiologists and radiation oncologists [7,8].

There has been an increasing personalization of interventional radiotherapy treatment planning through the adoption of image-guided interventional radiotherapy. Growing evidence on dose escalation indicates that the greatest benefit of image-guided interventional radiotherapy occurs in patients with tumors larger than 5 cm at diagnosis [9]. Several studies have shown that pre-treatment tumor volume and tumor regression ratio after radio-chemotherapy are among the strongest predictors of local recurrence [10,11,12,13,14]. Nonetheless, there remains a need to identify biomarkers that can guide treatment planning objectives in image-guided interventional radiotherapy, as it is not yet clear whether all patients derive the same benefit from dose escalation [15,16,17].

The aim of our study was to implement a predictive MRI-based model capable of evaluating the relationship between tumor shrinkage and clinical outcomes.

## 2. Materials and Methods

### 2.1. Study Design and Endpoint

A retrospective analysis of 300 consecutive patients treated for locally advanced cervical cancer between January 2021 and July 2024 was performed. Eligibility criteria included (1) cervix cancer confirmed by biopsy; (2) preoperative evaluation comprising physical examination, cervical biopsy, MRI, and fluorodeoxyglucose positron emission tomography/computed tomography (PET/CT); (3) treatment involving radio-chemotherapy followed by interventional radiotherapy; (4) availability of MRI at diagnosis and before image-guided interventional radiotherapy; and (5) written informed consent. All procedures were conducted in accordance with the standards of the institutional and national research committees, as well as the ethical principles outlined in the Declaration of Helsinki. The primary endpoints of the study were loco-regional control rate and metastasis-free survival. The secondary endpoint was overall survival. Actuarial loco-regional control was calculated from the date of the end of definitive radiotherapy to the occurrence of disease relapse or progression within the treated radiotherapy field, or to the date of the last follow-up. Actuarial metastasis-free survival was measured from the date of the end of definitive radiotherapy to the date of disease progression outside the treated field or to the last follow-up. Actuarial overall survival was defined as the time interval between the date of the end of definitive radiotherapy and either death from any cause or the last follow-up. Patients were evaluated weekly during treatment. Once treatment was completed, patients underwent scheduled surveillance every 6 months for the first 5 years (clinical examinations, MRI, PET-CT, and complete blood count) and then every 12 months.

### 2.2. Procedures

This study was performed within the framework of the COnsortium for BRachytherapy Data Analysis initiative, following its standardized guidelines. Collected data encompassed patient demographics, histologic subtype, radiotherapy technical and dosimetric parameters, treatment-related toxicities (acute and late), follow-up duration, and clinical outcomes.

### 2.3. External Beam Radiotherapy and Image-Guided Interventional Radiotherapy

External beam radiotherapy was delivered to a total dose of 45 Gray (Gy) in 25 fractions over five weeks using volumetric-modulated arc therapy. Involved lymph nodes received a simultaneous integrated boost to 55 Gy in 25 fractions for pelvic nodes and 57.5 Gy in 25 fractions for para-aortic nodes. Concurrent chemotherapy consisted of five weekly doses of cisplatin at a dose of 40 mg/m^2^.

All patients received image-guided high-dose-rate interventional radiotherapy, delivering a total dose of 28 Gy over four high-dose-rate fractions to the high-risk clinical target volume (HR-CTV) and a total equivalent dose in 2 Gy fractions of 60 Gy to the intermediate-risk clinical target volume (IR-CTV). Planning aims included a cumulative EQD2 of >90 Gy (with an upper limit of <95 Gy) for D90 of the HR-CTV, >75 Gy for D98 of the HR-CTV, >95 Gy for D98 of the residual gross tumor volume (GTVres), and >60 Gy for D98 of the IR-CTV. Limits for prescribed dose were defined as >85 Gy for D90 of the HR-CTV and >90 Gy for D98 of the GTVres.

Pre-interventional radiotherapy MRI was performed for all patients one week before interventional radiotherapy implant with a 1.5 tesla MRI scanner (Echospeed Horizon and Infinity, General Electric Healthcare, GE), including conventional MRI and diffusion-weighted imaging sequences. Conventional sequences included axial T2- and T1-weighted fast spin-echo imaging, as well as high-resolution fast spin-echo T2-weighted images acquired in multiple planes (sagittal, oblique axial), oriented perpendicular to the long axis of the cervix. Diffusion-weighted imaging was performed in the same orientation as the oblique axial fast spin-echo T2-weighted images, using a single-shot diffusion-weighted echo-planar sequence with two b-values (0 and 1000 s/mm^2^) [5]. The OncentraBrachy Treatment Planning System (Elekta, Sweden) was used to generate the four CT-based treatments. Total equivalent dose in 2 Gy fractions was calculated by combining contributions from both external beam radiotherapy and interventional radiotherapy, using an α/β ratio of 10 Gy for tumor tissue and 3 Gy for organs at risk.

Contouring included the gross tumor volume, high-risk clinical target volume, and intermediate-risk clinical target volume. The gross tumor volume comprised all visible tumors at the time of image-guided interventional radiotherapy, as determined by clinical assessment and MRI. The high-risk clinical target volume included the residual tumor along with adjacent cervical tissues. The intermediate-risk clinical target volume covered initial macroscopic disease with, at most, residual microscopic disease at the time of interventional radiotherapy. The bladder, rectum, and small bowel were considered organs at risk, and they were contoured and considered during planning. After applicator reconstruction, dwell positions and times were optimized manually to achieve the prescribed dose while respecting organ constraints.

### 2.4. Statistical Analysis

Variables derived from MRIs, including tumor volume at diagnosis, tumor volume at the time of pre-interventional radiotherapy MRI, and tumor volume reduction rate, were collected. Associations between MRI-derived volumetric variables and oncological outcomes were initially explored using descriptive and univariate analyses. The analysis was restricted a priori to three predefined imaging-derived parameters: tumor volume at diagnosis, tumor volume at the time of pre-interventional radiotherapy MRI, and tumor volume reduction rate. Time-to-event outcomes, including loco-regional relapse and distant metastases, were analyzed using Cox proportional hazards regression models. Follow-up time was defined as the interval between the end of definitive radiotherapy and the occurrence of the event of interest or the last available follow-up. Hazard ratios (HRs) with 95% confidence intervals were estimated. The Cox models were used to quantify the association between MRI-derived volumetric parameters and oncological outcomes within an exploratory analytical framework. In addition to MRI-derived volumetric variables, additional relevant factors (e.g., FIGO stage, age, overall treatment time, chemotherapy administration, histology) were explored to verify the consistency of the dataset with previously reported prognostic factors in cervical cancer. The number of covariates included in the multivariable models was selected according to the number of observed events to ensure model stability.

### 2.5. Nomogram Development

Subsequently, univariate Cox proportional hazards regression analyses were performed for each selected predictor to quantify its association with time-to-event outcomes. These outcomes correspond to loco-regional control and metastasis-free survival. Predictors that remained statistically significant during univariate analysis were included in multivariable Cox proportional hazards models to identify independent predictors of loco-regional relapse and distant metastases. Hazard ratios (HRs) with 95% confidence intervals (CIs) were reported.

All statistical analyses were conducted using standard statistical software. All tests were two-sided, and a *p*-value < 0.05 was considered statistically significant. Model estimates represent the relative contribution of each predictor to the risk of the outcomes as derived from Cox regression models. Based on the results of the multivariable Cox regression analyses, a nomogram was developed. The nomogram was developed as a graphical representation of the relative contribution of each predictor and to facilitate interpretation of the multivariable Cox models, rather than to provide direct individualized clinical risk prediction. Regression coefficients derived from the multivariate analyses were converted into a graphical scoring system to facilitate interpretation of the relative contribution of each predictor. Each predictor was assigned a weighted number of points proportional to its regression coefficient, and the sum of all points corresponded to an estimated risk score reflecting the relative contribution of each predictor to the outcomes.

A web-based interactive version of the nomogram was implemented using a stand-alone HyperText Markup Language and JavaScript framework, enabling real-time visualization of model outputs for exploratory purposes only. The tool is not intended for direct clinical decision-making. The nomogram provides an exploratory visualization of the combined effect of MRI-derived volumetric parameters on oncological outcomes, relying exclusively on the variables included in the final Cox models.

### 2.6. Statistical Validation

Model performance was assessed using Harrell’s concordance index (C-index), which is appropriate for time-to-event models. Internal validation was performed using a bootstrap resampling approach to assess the stability of the Cox regression models and to estimate optimism-corrected performance.

## 3. Results

### 3.1. Patient, Tumor, and Treatment Characteristics

Staging MRI and pre-interventional radiotherapy pelvic MRI of 300 patients with locally advanced cervical cancer were analyzed between January 2021 and July 2024. The patient and tumor characteristics are listed in Table 1. Median age at diagnosis was 53 years (range: 25–85). Most patients had International Federation of Gynecology and Obstetrics stage IIIC1 cancer (N  =  154, 51.3%) and squamous carcinoma (N  =  255, 85%)”. All patients received 45 Gy pelvic external beam radiotherapy, and 237 patients (82%) were treated with a simultaneous integrated boost on pelvic positive nodes, and 96 patients (33%) on para-aortic nodes. Two hundred and ninety-five patients received concurrent chemotherapy. Among them, 275 patients (91.6%) were treated with cisplatinum-based chemotherapy, 9 patients (3%) received carboplatin alone, considering the age or general status of the patient, and 11 (3.7%), a combination of cisplatin and fluorouracil. All patients underwent intracavitary or hybrid (intracavitary plus interstitial) interventional radiotherapy (total dose 28 Gy over four fractions). Median tumor size at diagnosis was 69 cm^3^ (range 0.63 cm^3^–1029.6 cm^3^), and at the time of pre-interventional radiotherapy, pelvic MRI was 2.2 cm^3^ (range 0 cm^3^–142.29 cm^3^). Among patients experiencing recurrence, the median time to T recurrence from the end of radiotherapy was 10.3 months (interquartile range (IQR): 7.9–13.4, range: 4.5–21.6 months), to nodal recurrences was 6.2 months from the end of radiotherapy (IQR: 3.8–9.7, range: 5.5–33.1 months) and time to metastasis was 7.0 months (IQR: 4.4–15.6, range: 5.5–33.1 months).

### 3.2. Treatment Outcomes

At a median follow-up of 22 months (range 6–56), 29 patients (9.9%) had died, 205 patients (70.2%) were alive without disease, and 58 patients (19.9%) were alive with disease. The median overall treatment time was 53 days (40–90 days). During follow-up, local recurrence occurred in 17 patients (5.7%), regional nodal recurrence in 49 patients (16.3%), and distant metastases in 69 patients (23.0%). Some patients experienced multiple sites of recurrence. The actuarial local control was 95.3% at 1 year and 94.3% at 2 years. Two-year overall survival, metastasis-free survival, and local–regional control were 92.8%, 86.3%, and 83.7%, respectively.

### 3.3. Statistical Analysis Results

In the univariate Cox analysis, tumor volume at diagnosis was associated with loco-regional relapse (HR = 1.004, 95% CI: 1.000–1.007, *p* = 0.031) and distant metastases (HR = 1.09, 95% CI: 1.04–1.15, *p* < 0.001), and remained independently associated in multivariate models (loco-regional relapse: HR = 1.003, 95% CI: 1.000–1.006, *p* = 0.041; distant metastases: HR = 1.08, 95% CI: 1.03–1.14, *p* < 0.001).

Tumor volume at the time of pre-interventional radiotherapy MRI was similarly associated with both endpoints during univariate (loco-regional relapse: HR = 1.006, 95% CI: 1.002–1.011, *p* = 0.004; distant metastases: HR = 1.011, 95% CI: 1.005–1.017, *p* < 0.001) and multivariate analysis (loco-regional relapse: HR = 1.004, 95% CI: 1.000–1.009, *p* = 0.038; distant metastases: HR = 1.007, 95% CI: 1.001–1.013, *p* = 0.019).

Tumor volume reduction rate was inversely associated with both outcomes during univariate (loco-regional relapse: HR = 0.982, 95% CI: 0.969–0.996, *p* = 0.011; distant metastases: HR = 0.975, 95% CI: 0.960–0.991, *p* = 0.002) and multivariate analysis (loco-regional relapse: HR = 0.987, 95% CI: 0.973–0.999, *p* = 0.041; distant metastases: HR = 0.981, 95% CI: 0.965–0.998, *p* = 0.029). A reduction rate ≥ 60% was associated with lower risk of both loco-regional relapse and metastatic progression (Table 2).

Regarding model performance, multivariate Cox models demonstrated moderate discrimination, with C-index values of 0.70 for loco-regional relapse and 0.77 for distant metastasis. Internal validation using bootstrap resampling confirmed the stability of these estimates.

Prognostic variables were also evaluated within the Cox regression framework. Age showed a borderline association with overall survival (HR = 1.020, 95% CI: 0.999–1.042, *p* = 0.062). Increasing FIGO stage was significantly associated with a higher risk of loco-regional recurrence (HR 1.42, 95% CI 1.05–1.93, *p* = 0.021), distant metastases (HR 1.67, 95% CI 1.12–2.49, *p* = 0.012), and worse overall survival (HR 1.58, 95% CI 1.10–2.28, *p* = 0.015). Overall treatment time > 50 days was significantly associated with worse overall survival (HR = 1.036, 95% CI: 1.008–1.064, *p* = 0.010). Non-squamous histology was associated with a higher risk of distant metastases (HR 1.82, 95% CI 1.01–3.27, *p* = 0.047), while no significant association was observed with loco-regional recurrence (HR 1.36, 95% CI 0.78–2.39, *p* = 0.28). A trend toward worse survival was observed (HR 1.64, 95% CI 0.95–2.83, *p* = 0.074), although this did not reach statistical significance.

To further evaluate the independent prognostic contribution of MRI-derived volumetric parameters, additional multivariate Cox regression models were fitted, including FIGO stage and histological subtype (Table 3 and Table 4). In the adjusted model for loco-regional relapse (Table 3), baseline tumor volume (HR = 1.001, 95% CI: 0.998–1.003, *p* = 0.04) and tumor volume reduction rate (HR = 0.997, 95% CI: 0.982–1.007, *p* = 0.03) were independently associated with outcome after adjustment. In the adjusted model for distant metastases (Table 4), baseline tumor volume remained independently associated with metastatic risk (HR = 1.004, 95% CI: 1.002–1.005, *p* < 0.001), and tumor volume reduction rate retained an inverse association (HR = 0.991, 95% CI: 0.982–0.999, *p* = 0.04). Cox proportional hazards regression models were used as the primary analytical framework to evaluate time-to-event outcomes. The observed associations consistently demonstrated that baseline tumor volume was associated with increased risk, while tumor volume reduction rate was inversely associated with oncological outcomes.

An online nomogram (eNomogram) calculator has been developed to estimate personalized outcome probabilities. The tool can be accessed at https://circe-nomogram.com.

## 4. Discussion

Our results demonstrate that small baseline tumor volume, small residual tumor volume before interventional radiotherapy, and a high tumor volume reduction rate, particularly ≥60%, were associated with a lower risk of loco-regional relapse and distant metastases. The model was intentionally restricted to imaging-derived parameters to ensure reproducibility, minimize subjectivity, and facilitate integration into routine radiotherapy practice.

Baseline tumor volume at the time of diagnostic MRI emerged as a strong predictor of treatment outcome. This finding is consistent with multiple studies demonstrating that larger tumor volumes are associated with poorer local–regional control and survival in cervical cancer [12,18,19]. Ángeles-Martínez et al. reported that baseline MRI tumor volume was significantly associated with disease failure and survival in patients treated with radio-chemotherapy, supporting tumor volume as a surrogate marker of intrinsic tumor aggressiveness [12]. Similar observations have been reported by Mayr et al., who demonstrated that initial tumor volume was a major determinant of outcome and was associated with both pelvic failure and distant spread [19]. Given the intrinsic relationship between baseline tumor volume, residual tumor volume, and volume reduction rate, potential collinearity among volumetric variables was assessed and found to be acceptable for multivariable modeling.

In the present cohort, the median HR-CTV D90 EQD2 was 90 Gy, in line with EMBRACE-based dose prescription strategies. In the EMBRACE I study, the median HR-CTV D90 EQD2 was 89.7 Gy in the overall cohort and 86.1 Gy in patients who developed local failure, with a reported 5-year local control of 92%. EMBRACE II established planning aims recommending an HR-CTV D90 above 90 Gy (target 90–95 Gy EQD2) and an IR-CTV D98 above 60 Gy. In our series, actuarial local control was 95.3% at 1 year and 94.3% at 2 years. Although follow-up in our cohort is shorter, the HR-CTV dose delivered and the observed local control rates appear consistent with the outcomes reported in the EMBRACE experience [20,21].

Beyond baseline imaging, our results highlight the prognostic importance of residual tumor volume assessed by pre-interventional radiotherapy MRI. Previous studies evaluating mid-treatment or pre-interventional radiotherapy MRI have shown that persistent tumor volume during radio-chemotherapy is associated with inferior local control and survival. Sun et al. demonstrated that residual tumor volume at the time of mid-treatment MRI was an independent predictor of overall survival and disease progression, emphasizing the relevance of early response assessment [22]. These findings are consistent with our multivariate analysis, in which RM pre-interventional radiotherapy remained independently associated with both loco-regional relapse and metastatic risk.

Previously published prognostic models in locally advanced cervical cancer rely on static baseline parameters, such as age, FIGO stage, non-squamous histology, and overall treatment time. Prognostic variables have also been evaluated within the Cox regression framework. Increasing FIGO stage was significantly associated with a higher risk of loco-regional recurrence, distant metastases, and worse overall survival. Longer overall treatment time and non-squamous histology were significantly associated with worse overall survival and a higher risk of distant metastases, respectively. These results confirm the well-established prognostic impact of disease stage [21,23,24,25,26]. Age showed a borderline association with overall survival. Previous cervical cancer series have also reported poorer survival in older patients [27,28].

Several MRI-based studies have demonstrated the prognostic value of baseline tumor volume alone, showing its association with local control, progression, and survival [12,18,19]. However, these approaches do not account for early treatment response and tumor dynamics during RCT. Other models have incorporated mid-treatment or post-external beam radiotherapy imaging to refine risk stratification. Studies by Sun et al. [22] and Mayr et al. [11] highlighted the prognostic relevance of residual tumor volume or early regression assessed during treatment. Nevertheless, these models typically evaluated individual parameters in isolation and were not translated into integrated multivariable frameworks. In the present study, we combined three longitudinal MRI-derived parameters—tumor volume at diagnosis, residual tumor volume before interventional radiotherapy, and tumor volume reduction rate—within a single modeling approach. This strategy captures both intrinsic tumor burden and early treatment response, providing a more comprehensive representation of disease behavior than baseline or single time-point assessments alone.

In contrast to previously published models that predominantly combine clinical and pathological variables or rely on static imaging features [29,30], the present model focuses exclusively on imaging-derived biomarkers obtained within standard MRI workflows routinely used in image-guided interventional radiotherapy. This approach may enhance consistency and reproducibility across clinical settings, although its applicability requires further validation.

Although the discriminative performance of the model is moderate and comparable to that reported in other prognostic studies, its main contribution lies in the integration of dynamic volumetric response parameters within a single multivariable framework. A graphical representation of the model was developed to facilitate interpretation of the multivariable associations. While similar approaches in the literature have proposed nomogram-based models for risk estimation [31,32], the present model should be interpreted as exploratory. It is not intended for direct clinical application, and its performance requires confirmation in external, independent cohorts before any potential use in clinical decision-making.

This study is based on a large and homogeneous cohort of patients with locally advanced cervical cancer treated with a standardized radio-chemotherapy and image-guided interventional radiotherapy protocol at a high-volume center. A major strength is the integration of longitudinal MRI-derived volumetric biomarkers, enabling response-oriented prognostic stratification beyond conventional staging. The use of multivariable modeling with internal bootstrap validation supports the robustness of the findings, although external validation remains essential. Potential multicollinearity among volumetric variables was formally assessed and found to be acceptable.

Several limitations should be acknowledged. The retrospective design may be associated with selection bias and unmeasured confounding. The models were developed using data from a single institutional workflow, which may limit generalizability. Although internal validation was performed, external validation in independent cohorts is required before any potential clinical application. In addition, the model included only imaging-derived variables and lacked biological or molecular factors, and the relatively limited follow-up may have underestimated late events. The present model should be interpreted as exploratory. The lack of external validation, the retrospective design, and the limited follow-up preclude its immediate use as a clinical decision-support tool. Instead, the model should be considered hypothesis-generating and may serve as a basis for future prospective validation studies. Time-to-event analyses were performed using Cox proportional hazards models to appropriately account for variable follow-up duration and censoring. The predictive model was intentionally restricted to imaging-derived variables and did not incorporate established clinical prognostic factors. Although this approach allowed the evaluation of the independent prognostic value of MRI-derived tumor response parameters, integrated models combining clinical and imaging variables may further improve predictive performance. MRI-derived volumetric parameters may be associated with early risk stratification in patients with locally advanced cervical cancer treated with radio-chemotherapy and image-guided interventional radiotherapy. However, the proposed model requires external, multi-institutional validation and should be considered as a foundation for future studies integrating imaging with clinical and molecular biomarkers.

## 5. Conclusions

Our findings demonstrate that tumor volume at diagnosis, residual volume at the time of pre-interventional radiotherapy imaging, and reduction rate are independent predictors of loco-regional relapse and distant metastases. In this context, early identification of patients at higher risk of relapse or metastases may support treatment intensification strategies or closer surveillance. Our objective was not to replace existing clinical prognostic systems but to explore whether early volumetric response assessed by MRI could independently stratify patient risk. Clinical variables may be integrated into future expanded models following external validation.

## Figures and Tables

**Table 1:** Patient and tumor characteristics.

Number of patients	300
Median age	53 (25–85)
FIGO Stage	
IB1/2	4 (1.3%)
IIA	5 (1.7%)
IIB	34 (11.3%)
IIIA	2 (0.7%)
IIIB	4 (1.3%)
IIIC1	154 (51.3%)
IIIC2	59 (19.7%)
IVA	25 (8.3%)
IVB	13 (4.3%)
Histology	
Squamous cell carcinoma	255 (85%)
Adenocarcinoma	35 (11.7%)
Other	10 (3.3%)
Chemotherapy	
Yes	CDDP 275 (91.6%)
	AUCC 9 (3%)
	PLAFUR 11 (3.7%)
No	5 (1.7%)
Median tumor size at diagnosis	69.43 cm^3^ (range 0.63 cm^3^–1029.6 cm^3^)
Median pre-IRT pelvic MRI	2.20 cm^3^ (range 0 cm^3^–142.29 cm^3^).
Median reduction rate	96.49% (range 0–100)

Abbreviation: AUCC: carboplatin; CDDP: cisplatin; cm: centimeters; FIGO: International Federation of Gynecology and Obstetrics; MRI: magnetic resonance imaging; PLAFUR: cisplatin and fluorouracil.

**Table 2:** Univariate and multivariate analysis.

Loco-Regional Control				
Variable	Univariate HR (95% CI)	*p*-Value	Multivariate HR (95% CI)	*p*-Value
MRI diagnosis (cm^3^)	1.004 (1.000–1.007)	0.031	1.003 (1.000–1.006)	0.041
MRI pre-IRT (cm^3^)	1.006 (1.002–1.011)	0.004	1.004 (1.000–1.009)	0.038
Tumor reduction (%)	0.982 (0.969–0.996)	0.011	0.987 (0.973–0.999)	0.041
Metastasis				
MRI diagnosis (cm^3^)	1.09 (1.04–1.15)	<0.001	1.08 (1.03–1.14)	<0.001
MRI pre-IRT (cm^3^)	1.011 (1.005–1.017)	<0.001	1.007 (1.001–1.013)	0.019
Tumor reduction (%)	0.975 (0.960–0.991)	0.002	0.981 (0.965–0.998)	0.029

Abbreviation: CI: confidence interval; IRT: interventional radiotherapy; MRI: magnetic resonance imaging.

**Table 3:** Cox proportional hazards regression analysis for loco-regional relapse.

Variable	Hazard Ratio (95% CI)	*p*-Value
MRI diagnosis (cm^3^)	1.001 (0.998–1.003)	0.04
MRI pre-IRT (cm^3^)	1.004 (0.989–1.018)	0.63
Tumor reduction (%)	0.997 (0.982–1.007)	0.03

Abbreviations: CI, confidence interval; HR, hazard ratio; IRT: interventional radiotherapy; MRI, magnetic resonance imaging.

**Table 4:** Cox proportional hazards regression analysis for distant metastases.

Variable	Hazard Ratio (95% CI)	*p*-Value
MRI diagnosis (cm^3^)	1.004 (1.002–1.005)	<0.001
MRI pre-IRT (cm^3^)	0.988 (0.972–1.004)	0.129
Tumor reduction (%)	0.991 (0.982–0.999)	0.04

Abbreviations: CI, confidence interval; HR, hazard ratio; IRT: interventional radiotherapy; MRI, magnetic resonance imaging.

## Data Availability

The data presented in this study are available on reasonable request from the corresponding author.
